# Influence of Ti on the Tensile Properties of the High-Strength Powder Metallurgy High Entropy Alloys

**DOI:** 10.3390/ma13030578

**Published:** 2020-01-26

**Authors:** Igor Moravcik, Stepan Gamanov, Larissa Moravcikova-Gouvea, Zuzana Kovacova, Michael Kitzmantel, Erich Neubauer, Ivo Dlouhy

**Affiliations:** 1 Institute of Materials Science and Engineering, Brno University of Technology, Technicka 2896/2, 61669 Brno, Czech Republic; gamanov@ipm.cz (S.G.); gouvea@fme.vutbr.cz (L.M.-G.); dlouhy@fme.vutbr.cz (I.D.); 2 Institute of Physics of Materials of the Czech Academy of Sciences, Žižkova 513/22, 61600 Brno, Czech Republic; 3 RHP-Technology GmbH, Forschungs- und Technologiezentrum, 2444 Seibersdorf, Austria; zuzana.kovacova@rhp-technology.com (Z.K.); michaelkitzmantel@rhp-technology.com (M.K.); erichneubauer@rhp-technology.com (E.N.)

**Keywords:** multi principal element alloy, tensile strength, fracture, ductility, powder

## Abstract

The focus of this study is the evaluation of the influence of Ti concentration on the tensile properties of powder metallurgy high entropy alloys. Three Ni_1.5_Co_1.5_CrFeTi_X_ alloys with X = 0.3; 0.5 and 0.7 were produced by mechanical alloying and spark plasma sintering. Additional annealing heat treatment at 1100 °C was utilized to obtain homogenous single-phase face centered cubic (FCC) microstructures, with minor oxide inclusions. The results show that Ti increases the strength of the alloys by increasing the average atomic size misfit i.e., solid solution strengthening. An excellent combination of mechanical properties can be obtained by the proposed method. For instance, annealed Ni_1,5_Co_1,5_CrFeTi_0.7_ alloy possessed the ultimate tensile strength as high as ~1600 MPa at a tensile ductility of ~9%, despite the oxide contamination. The presented results may serve as a guideline for future alloy design of novel, inclusion-tolerant materials for sustainable metallurgy.

## 1. Introduction

The equiatomic high entropy alloys (HEA) designed by Yeh and Cantor [1,2] are a new class of metallic materials, composed of at least five elements and with a common feature being a lack of single major element. Despite some ongoing arguments regarding the origins (as well as the existence) of their special properties [3], the mentioned core idea spawned a new direction in the development of metallic, as well as non-metallic materials [4,5]. Even though considerable resources has been dedicated to the study of a vast range of HEA properties, phase compositions as well as guiding principles for further alloy design [6,7,8,9], the properties of most HEAs are still not competitive in comparison to more classic materials [3,10]. One of the biggest issues with HEAs is the vast compositional space resulting from inherent chemical complexity, in which it is extremely hard to choose the compositions [11]. On top of this, the competitiveness of any selected composition is a matter of a combination of several properties, not strictly constrained to combination of strength and ductility [12,13], as it may seem from current studies. One of the new HEA alloy systems which possess a very interesting combination of mechanical properties, oxidation and corrosion resistance combined with exceptional wear resistance [14,15,16,17,18], is Ni_1.5_Co_1.5_CrFeTi_X_. It exhibits a very high mechanical strength and ductility in a solutionized (single-phase FCC) state [17], while it can be heat treated to contain intermetallic strengthening phases. Its strength in single-phase state is derived from relatively high atomic size misfit *δ* (5.04 % for X = 0.5) of its elements [15], which is a measure of the extent of solid solution strengthening [19] i.e., the magnitude of critical resolved shear stress required for the dislocation movement. The atomic size difference *δ* based on the Hume—Rothery rules is defined as [20]:

(1) $$\delta =100\sqrt{\sum_{i=1}^{n}c_{i}{\left(1-\frac{r_{i}}{\bar{r}}\right)}^{2}}.$$

In this equation, *c*_i_ is the atomic concentration (molar fraction), *r*_i_ presents the atomic radius of i-th element and $\bar{r}$ is the average atomic radius $\bar{r}=\sum_{i=1}^{n}c_{i}r_{i}$ of a given system.

The difference between the atomic sizes of Ni, Co, Cr and Fe elements present in Ni_1.5_Co_1.5_CrFeTi_X_ HEA are relatively small [21,22]. The only exception is Ti, which possess much larger atoms, with respect to the latter. It is therefore intuitive that Ti concentration will largely dictate the extent of solid solution strength, and it would be advisable to keep it as high as possible. On the other hand, the binary mixing enthalpy ΔH_AB_ of Ti-Ni pair (−35 kJ/mol) and Ti-Co pair (−35 kJ/mol) is much more negative than for the other possible atomic pairs (minimum −7 kJ/mol for Ni-Cr) [21,23]. This means that increasing the Ti concentration will inevitably result in the thermodynamic drive towards the formation of intermetallic phases—decreasing the solid solution strengthening effect. To probe the effect of Ti concentration change on the mechanical properties as well as phase composition and Ti partitioning effects, the Ni_1,5_Co_1,5_CrFeTi_X_ alloys have been tested. Altogether, three different HEAs with Ti atomic ratios; x = 0.3, 0.5 and 0.7 were prepared. To prevent the segregation effects which may occur during the casting of such complex systems [24] and formation of coarse-grained microstructures, an alternative powder metallurgical manufacturing route of mechanical alloying (MA) and spark plasma sintering (SPS) was used. The latter process enables us to produce fine-grained materials and to study the properties without the need for time- and energy-consuming additional hot and cold-working processes, which are sometimes required for cast materials [15,25].

## 2. Materials and Methods

Three alloys with chemical composition of Ni_1.5_Co_1.5_Cr_1_Fe_1_Ti_x_ (where x = 0.3; 0.5; 0.7) were prepared by a powder metallurgy route and will be further referred to as Ti0.3, Ti0.5 and Ti0.7, respectively. The target composition in atomic % are Ni_28_Co_28_Cr_19_Fe_19_Ti_6_, Ni_27_Co_27_Cr_18_Fe_18_Ti_9_ and Ni_26_Co_26_Cr_17_Fe_17_Ti_12_ for Ti0.3, Ti0.5 and Ti0.7 alloys, respectively (also presented latter is Section 3.3), whereas, in weight %, the compositions are Ni_30_Co_29_Cr_19_Fe_19_Ti_5_, Ni_29_Co_29_Cr_17_Fe_18_Ti_8_ and Ni_28_Co_28_Cr_16_Fe_17_Ti_11_ in the same order.

It should be noted that the conditions used for mechanical alloying and spark plasma sintering are similar to our previous publication [15] on similar HEA. Alloys were prepared from commercial-grade purity, whereas the information on the powders given in Table 1. Powders were milled with 10:1 ball to powder weight ratio using balls with 15 mm and 20 mm diameter (1:1 ratio). Each starting powder mixture weighted 100 g. A ball and powder mixture was introduced into a steel milling container together. A milling container was then sealed and flushed with N_2_ gas to limit oxidation during the milling process. Dry milling was performed in Pulverisette 6 ball mill (Fritsch GmbH, Idar-Oberstein, Germany) with milling speed of 250 rounds per minute for 35 h of milling (30 min pauses after each hour of milling were used to prevent overheating). In the end of the dry-milling process, powders were further wet-milled for 1 h with 100 mL of ethanol to remove powders stuck to milling balls. Extracted powders were then filtered and dried. Metallographic specimens were prepared from powders and bulk materials were prepared by standard metallographic procedures. Energy-dispersive X-ray spectroscopy (EDS) analyses were performed on every powder to ensure its chemical homogeneity before the SPS process.

MA powders were subsequently sintered by the SPS method at RHP-Technology GmbH (city, country) into the form of 5 mm thick cylinders with 36 mm diameter using graphite dies. To prevent powder contamination by carbon, graphite dies were coated prior to sintering with chemically inert boron nitride. Sintering temperature of 1150 °C measured by thermocouple inside the die, with 30 MPa of pressure and 10 min. dwell time at sintering temperature was used. The heating rate of 100 °C/min. was used up to a temperature of 1000 °C. The heating rate was then reduced to 50 °C/min. up to 1150 °C/min. The whole process was performed in a vacuum atmosphere. At the end of sintering, the setup was left to naturally cool down, until it was opened at ~200 °C. Sintered samples were then annealed at 1100 °C for 24 h and water cooled, to obtain microstructural relaxation, homogenization and pore closure (in case of Ti0.3 alloy). These materials will be henceforth referred to as Ti0.3A, Ti0.5A and Ti0.7A, respectively. The porosity, as well as particle size measurement, was performed with image analysis in ImageJ software on images taken with optical microscope (Olympus GX51, Tokyo, Japan). Average grain size measurement was performed with a linear intercept method on FCC grains and oxide particle size was measured with special module embedded in ImageJ software. ULTRA PLUS SEM (Carl Zeiss AG, Oberkochen, Germany) and Lyra XMA FEG/SEM (TESCAN, Brno, Czech Republic) were used for SEM and energy-dispersive X-ray spectroscopy (EDS) analysis of bulk microstructures and fracture surfaces. A Smartlab instrument (Rigaku, Tokyo, Japan) with Co source was utilized for X-ray diffraction (XRD) analysis of phase composition and lattice parameters. A microhardness test with a Microharness tester LM247AT (Leco, St. Joseph, MI, USA) was carried out with 100 g load (HV0.1). Tensile tests were carried out on cylindrical tensile specimens with gauge length of 12.5 mm and diameter of 3.5mm were cut and machined from bulk materials. An Instron 8801 machine (Instron, Norwood, MA, USA) was used for tensile tests performed at room temperature and cross-head speed of 0.25 mm/min. Two tensile samples were tested for each of annealed TixA materials. Calculation of property diagrams (CALPHAD) were performed using Thermo-Calc software version 2019a (TCHEA3 database version 3.1, (Thermo-Calc, Solna, Sweden)). The thermodynamic modeling was performed for estimation of correct temperatures for annealing, as well as to get a better understanding of possible phase composition of the manufactured materials.

## 3. Results

### 3.1. Prepared Powder before SPS

**Figure 1:**
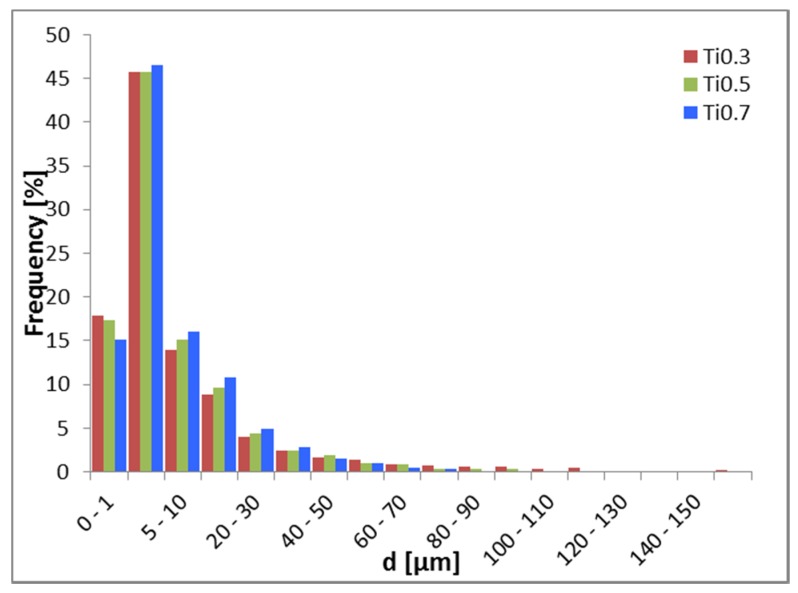
Histogram of powder particles distribution of Tix alloys. The largest fraction of particles have a size between 1 and 5 µm. Particles with size larger than 100 µm are significantly frequent only in the case of Ti0.3 powder.

Average particle size d of powders is given in Table 2. It is worth noting that each prepared powder had a large number of relatively small particles (above 75% of particles with d below 10 µm for all powders) and a low number of relatively large particles. This is especially evident in powder Ti0.3 with particles diameter up to 250 µm as can be seen in Figure 1. Therefore, Ti0.3 presents the coarsest powder among all of them, while Ti0.5 and Ti0.7 are relatively identical. The EDS analysis revealed homogenous chemical composition on powder particles; more precise analysis was later performed on sintered and annealed bulk samples.

### 3.2. Phase Diagram

**Figure 2:**
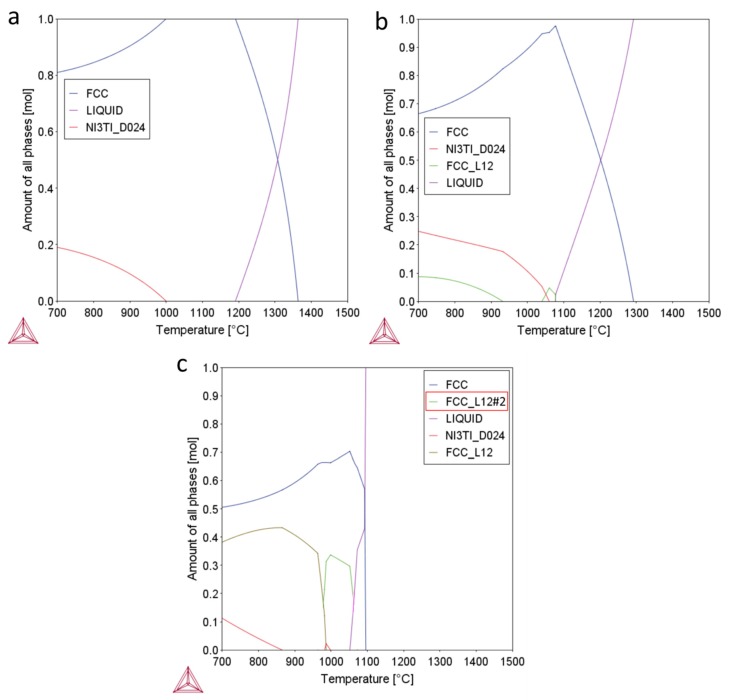
Results of the CALPHAD predictions of Ni1.5Co1.5CrFeTix alloys showing equilibrium phases and their respective fraction at different temperatures; (**a**) Ti0.3; (**b**) Ti0.5; (**c**) Ti0.7.

The results of the CALPHAD predictions of Ni_1.5_Co_1.5_CrFeTi_x_ alloys showing equilibrium phases are shown in Figure 2. It should be noted, in the start, that the accuracy of the CALPHAD predictions for such complex systems may not be perfect [26], and the real microstructures and phases fraction may show certain discrepancies. Nevertheless, it serves as useful general guidance for further phase characterization. The Ti0.3 material should be composed from a mixture the FCC solid solution phase and Ni_3_Ti (HCP DO_24_) ordered η phase, found commonly in Ni-base superalloys. The Ti0.5 alloy should contain an additional ordered (L_12_) FCC phase, precipitating at temperatures below ~900 °C. According to the prediction, this ordered FCC phase should have a (Ni,Co)_3_Ti chemistry. It has been shown before that the Ni_3_Ti ordered η phase and ordered FCC L_12_ phases show similar morphologies and they can coexist in the microstructures of Ni-base alloys [27], due to their close chemistry and crystallography. With increasing Ti concentration, the melting temperature is slightly decreasing (Figure 2c) in Ti0.7 alloy, while the phase composition should be identical to that of Ti0.5 alloy, but with increasing volume fraction of η and ordered FCC L_12_ phase. The ordered FCC L_12_#2 phase (marked by red rectangle in Figure 2c) has probably not formed in the structure and should be identical to FCC L_12_. It probably appeared due to an uncertainty in the calculation, which has been observed before [14,28]. At the processing temperatures to which the materials were subjected to (1150 or 1100 °C), the microstructures of the alloys should contain only single FCC phase. This single-phase microstructure should be mostly retained due to relatively fast cooling after SPS [14,15]. Despite this, some extent of second phase precipitation may occur, especially in Ti0.7 alloy. To make the microstructures more uniform, annealing treatment was used. After annealing at 1100 °C and water quenching, all materials should show single FCC phase, ensuing the highest extent of solid solution strengthening.

### 3.3. Microstructure and Phase Analysis after Sintering and Annealing

**Figure 3:**
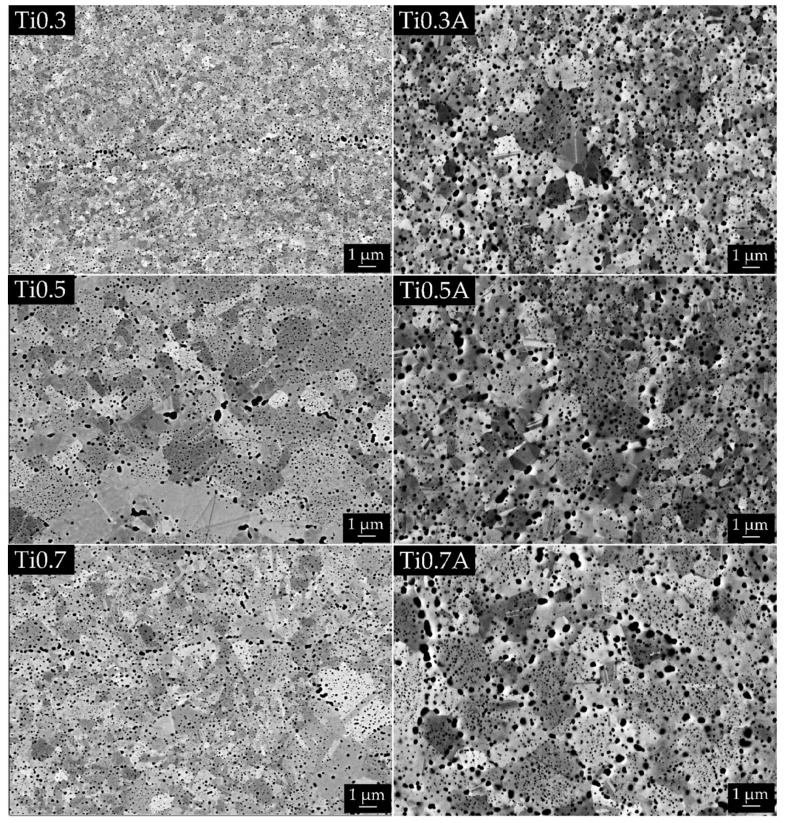
Structure of Tix alloys in sintered and annealed state, backscattered electron (BSE) SEM micrographs. It can be seen that FCC grains in all materials coarsened as well as the oxides (black dots). There are visible lines of coarsest oxides in the case of (**Ti0.3**) and (**Ti0.7**); these oxides probably copy the shape of original powder particles. There are also very fine particles inside the grains (best seen in case of (**Ti0.7A**); these particles are suspected to not be the oxides but intermetallic particles. Please note that the light grey–dark grey contrast between FCC grains is caused by different crystallographic orientation.

**Figure 4:**
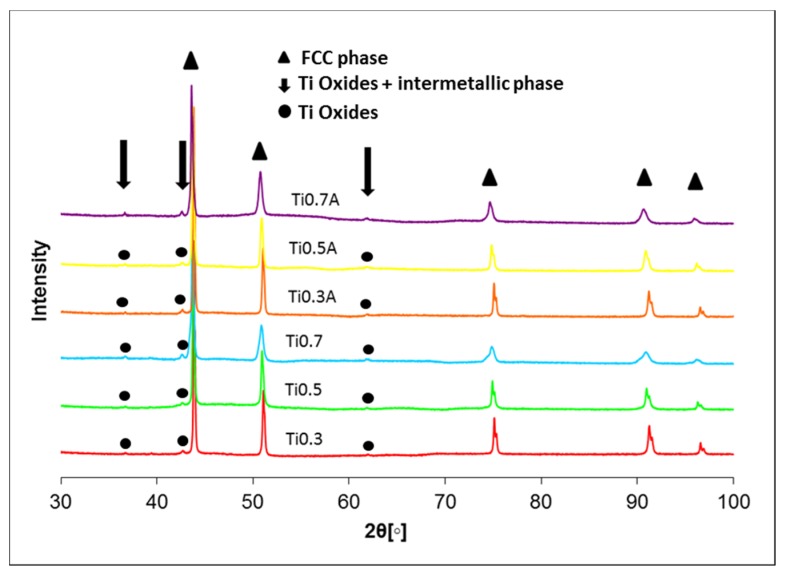
XRD spectrums of sintered an annealed Tix alloys.

**Figure A1:**
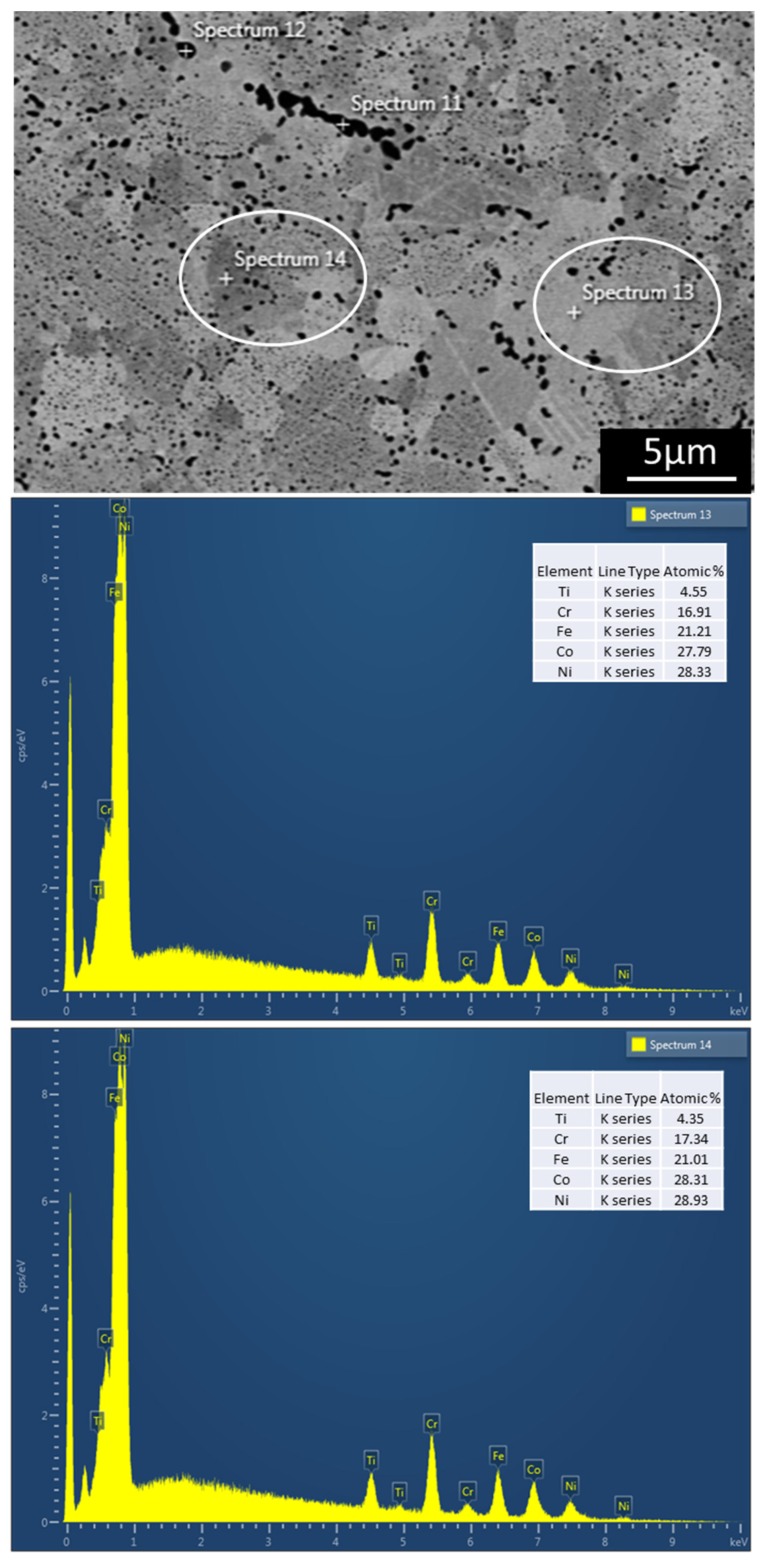
Representative EDS points in FCC matrix of Ti0.5 alloys in the SPS-ed state marked on the BSE micrograph. The presented points possess very close chemical composition despite the different shades of grey in the respective grains that spectras were taken from. Therefore, the contrast between different FCC grains in BSE mode is caused by differences in crystallographic orientation and not in chemistry.

**Figure A2:**
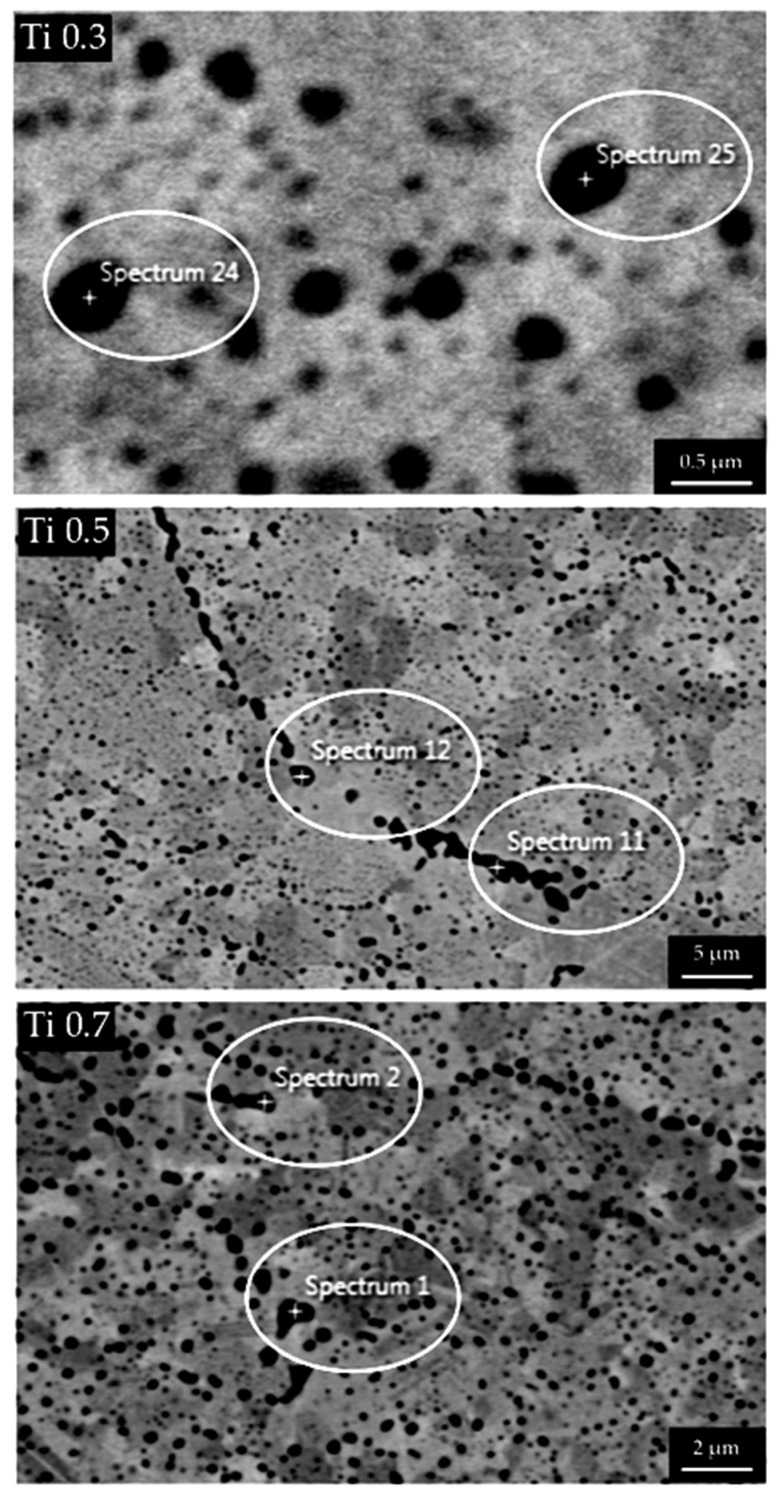
Representative EDS analysis of oxides on Tix alloys in SPS-ed state. The largest of the black dots were chose for point spectrum analysis to ensure that the majority of detected signal is coming from the particle and not from matrix below. The exact chemical compositions measured are presented in the Table A1 below. Micrograph of (**Ti0.5**) corresponds to an area also shown in Figure 5 in the main text.

Table 3 shows data acquired via image and XRD analysis of sintered and annealed alloys. Significant porosity was measured only for Ti0.3 alloy, which had the coarsest powder, while the rest of the alloys obtained full-density already after SPS. The lower porosity (at same sintering temperature) of Ti0.5 and Ti0.7 materials compared to Ti0.3 can be also caused by lowering of melting temperature by Ti, as calculated before. In regards to porosity, additional annealing was successful since it reduced porosity of Ti0.3 from 3.77% to 0.43%. The representative microstructures of all materials are presented in Figure 3. The grain size of all alloys in sintered state was very fine i.e., below 1.7 µm, whereas it grew after annealing up to 2.13 µm for the Ti0.7 alloy. Differences in shades of gray between different FCC grains in the BSE images of Figure 3 are caused by differences in crystallographic orientation, as shown in Appendix Figure A1, due to special setup used for the imaging of grains [29] without the need for electrochemical etching. A large fraction of oxides (visible as black dots) was present in all alloys even though the powders were prepared in an inert atmosphere. More detailed EDS analysis on the representative black particles present in all microstructures is given in the Appendix in Figure A2 and Table A1, proving that they pertain to oxides and not to porosity. It should be noted that the small size of the oxides combined with the resolution and insensitivity of EDS to lighter elements did not enable us to measure the character of oxides precisely. Even the largest of the oxides are still too small for EDS and the signal coming out of oxides is contaminated by the signal from the matrix below the particles (Table A1). However, it is clear that the oxides are mostly enriched in Ti elements. The high concentration of light Ti and O (compared to other present elements) causes them to appear dark compared to the matrix in back-scattered electron (BSE) images. This is different to the pores which also appear black, but in the secondary electron micrographs [30,31]. The powders of commercial-purity already possessed an oxide layer on their surfaces prior to mechanical alloying, which got dispersed into the powder particles during the latter process. In addition, the contamination of the powders was enhanced due to an additional milling step in ethanol. These oxides get trapped inside of the specimen during the SPS-densification process. The average size of the oxides was below 100 nm for all sintered alloys and between 150 and 175 nm for annealed alloys. It should be noted that the small size of the oxides combined with the resolution and insensitivity of EDS to lighter elements did not enable us to measure oxides character precisely. XRD analysis of the produced alloys shown in Figure 4 revealed that all SPS-ed alloys are dominantly composed of FCC solid solution phase (in agreement with the CALPHAD predictions) with a minor fraction of oxide phase. As mentioned before, the oxide phase is a result of powder surface contamination. However, due to a small fraction of this oxide phase(s) (~3.5%), it would be imprecise to conclude its true nature only from XRD. It corresponds to a Ti oxide, which can also contain small concentrations of other elements. The fraction of the secondary phase did not decrease after annealing at 1100 °C, which is caused by high thermal stability of the oxide. The only exception to this is the Ti0.7 alloy, which showed a decrease of the secondary phase after annealing. Therefore, it can be concluded that Ti0.7 alloy prior to annealing contained an additional fraction of intermetallic phase as predicted by the CALPHAD with the same peaks as the oxide phase, while a decrease in their intensity was observed after annealing. This tertiary intermetallic phase was not observed by SEM before due to its low fraction.

**Figure 5:**
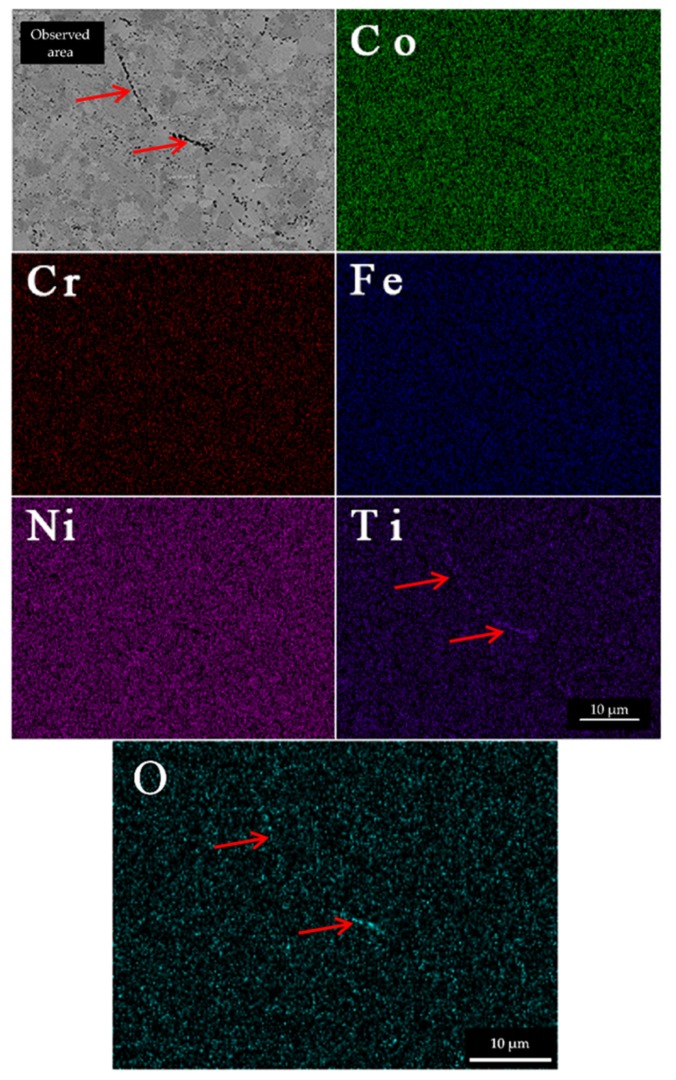
Representative EDS elemental maps of Ti0.5 alloy. The elements are evenly distributed in the alloy. The only exception is Ti, which concentrates in the oxide particles. The Ti- enriched oxide strings highlighted by red arrows denominate powder particle boundaries prior to SPS densification, but they are also present inside of former particles. The signal of O is strongest in the same regions as Ti, most noticeably in the region highlighted by red arrows of oxide strings.

EDS analysis of matrix of the Tix alloys are given in Table 4 with a representative EDS map in Figure 5 (including the oxygen map).

Statistical data on overall chemical compositions of Table 4 are composed of point EDS spectrums (points were always placed farther away from the oxides). The alloy compositions present a good fit to the target chemical compositions. The content of Ti in matrix of Ti0.7 alloy increased after annealing. This indirectly suggests us that some intermetallic compounds were present before annealing, as evidenced before by XRD. As these phases dissolved during annealing, the content of Ti in matrix increased. The elements in FCC phase of materials show even distribution (Figure 5), while only the Ti is localized also in the oxide particles (denoted by red arrow).

### 3.4. Mechanical Testing

The results of microhardness test are presented in Table 5 The Ni_1.5_Co_1.5_CrFeTi_X_ materials exhibit relatively high hardness values, due to very fine grain size and the presentation of hard particles. It should be noted that the influence of porosity on the hardness of the Ti0.3 materials was negligible, since the size of the micro-indents was significantly smaller than the size of the pores. Therefore, it was possible to measure hardness on areas with full-density at a considerable distance from the pores. As expected, the average hardness of the materials is increasing with increasing the Ti concentration in both conditions (SPS-ed and annealed). The annealing treatment decreased the average hardness and led to higher hardness uniformity (smaller values scatter). Compared to Ti0.3 and Ti0.5, the annealing of Ti0.7 sample led to much lower hardness decrease.

**Figure 6:**
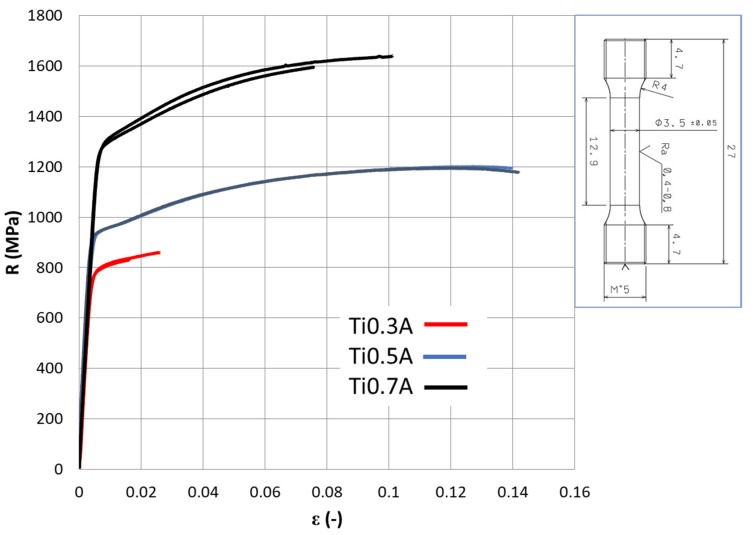
The tensile curves obtained from the Ni_1.5_Co_1.5_CrFeTi_X_ alloy specimens after annealing. The sample geometry is presented on the right-hand side, dimensions in mm.

The results of tensile tests are displayed in Figure 6 and Table 6 The worst values of tensile yield strength (R_p0.2_) of 781 MPa, ultimate tensile strength (R_m_) of 845 MPa and only ~2% of ductility (A_t_) were observed for the Ti0.3A samples due to premature fracture caused by porosity. On the other hand, full density samples Ti0.5A showed very good combination of Rp_0.2_ ≅ 930 MPa, R_m_ ≅ 1200 MPa and A_t_ ≅ 14%. Slight necking was also observed (plastic deformation after plastic instability threshold R_m_). The highest strength properties of Rp_0.2_ ≅ 1220 MPa, R_m_ ≅ 1680 MPa were measured for Ti0.7A material, with slightly lower ductility of A_t_ ≅ 9%. It is interesting to note that the highest strain hardening (R_m_–R_p0.2_) was observed in the strongest material Ti0.7A, which is contradictory to usual observations [32].

**Figure 7:**
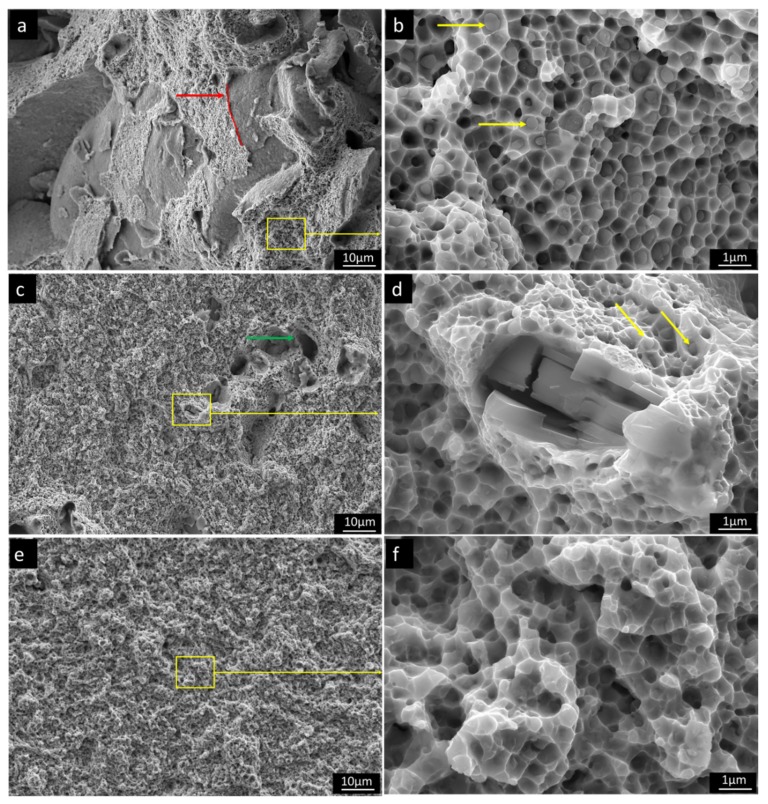
Fracture surfaces of Ni_1.5_Co_1.5_CrFeTi_X_A material; oxide particles inside the ductile dimples are marked by yellow arrows. (**a**,**b**) Ti0.3A, showing ductile dimples and residual porosity—the edge of the former pore is denoted by a red arrow and spline; (**c**,**d**) Ti0.5A showing ductile dimples and holes are denoted by green arrow with larger fractured oxide particles; (**e**,**f**) Ti0.7A exhibiting very flat ductile fracture surface and small dimples lacking oxide particles. Oxide particles inside the ductile dimples are marked by yellow arrows.

Figure 7 shows representative fracture morphologies of broken tensile specimens. All of the samples exhibited ductile fracture dimples with the size between of 200–800 nm. On fracture surface of Ti0.3A material, surfaces of pores present in the fracture surface of the samples are exposed, denoted by a red arrow with original pore surface (matrix-pore interphase) marked by red spline. Despite the low measured porosity in Ti0.3A alloy after annealing (<0.5%), the pores appeared on fracture surfaces of Ti0.3A in much larger contents. This happens because the forming crack tip is following areas with the largest porosity levels, as they locally act as stress concentrators. The fine oxide particles are found at the ductile dimple centers, which suggests their role as a nucleation size for dimple formation. In comparison to Ti0.3A, no porosity was observed on the fracture surface of Ti0.5A, which agrees well with its largest ductility. The dimple size of the Ti0.5A is slightly smaller with lower number density of oxides in their centers. The pore-like formations denoted by the green arrow (Figure 7c) correspond to areas with larger oxides that were pulled from the matrix during fracture process. Larger internally cracked oxide particles can also be observed on the fracture surfaces (Figure 7d). The fracture surface of the Ti0.7A material is much flatter with lower surface roughness compared to Ti05A material, corresponding to its lower ductility. This type of fracture corresponds to low-energy ductile tearing observed in severely strengthened ductile metals [33]. Ductile dimples of Ti0.7 material are largely free of oxides and very shallow, which suggest that dimple nucleation started inside the FCC matrix due to its higher strength and consequential lower plasticity, compared to Ti0.3A and Ti0.5A.

## 4. Discussion

Considering the influence of the Ti concentration on the properties of the milled powders, with its increasing concentration, the powder particle size is decreasing. This can be associated with increasing the intrinsic strength of the alloy by Ti. The extent of solid solution strengthening given approximately by atomic size difference in Table 7 is increasing with increasing Ti. This causes strengthening of the FCC matrix but decreases in its plasticity. Consequently, the powders have a lower tendency to agglomerate, resulting in smaller particle size.

The increase in the Ti concentration has a positive influence on the densification rate of Ni_1.5_Co_1.5_CrFeTi_X_ alloy (at the same sintering temperatures) since an alloy with the least Ti (Ti0.3) alloy showed the largest porosity after SPS. This can be caused by a decreasing of melting temperature by Ti (Figure 2), which promotes faster densification.

Considering only the comparison of TiXA alloys between each other, the increase in strength associated with grain boundary strengthening can be neglected due to a relatively narrow range of obtained grain sizes (from 1.3 μm to 2.13 μm in annealed state). In the same way, Orowan strengthening by the oxide inclusions can also be omitted since the volume fraction of oxides is similar (~3.5%) in all compositions. Consequently, the aforementioned increase of strength of the solid solution by Ti is directly observed from the increase of lattice parameter [34] from 3.58 Å in Ti0.3A material, to 3.6Å in Ti0.7A caused by Ti. This is in good agreement with the increasing hardness and tensile strength by Ti, especially in annealed conditions. On the other hand, the ductility of the materials seems to be decreasing with increasing Ti concentration. It should be noted that low ductility of the Ti0.3A material was caused by the porosity, rather than by the inherent properties of the FCC solid solution.

All the feedstock powders were probably contaminated on their surfaces by oxygen prior to MA. After the MA and SPS densification, even though it was problematic to measure their precise chemical composition, we can safely say that the oxides contained mostly Ti, due to its highest affinity to oxygen with respect to other present elements. This suggests that all the oxides of different elements recombined during MA (in situ) or subsequent SPS sintering into Ti-rich oxides. This also exhorts the possibility of modification of these oxides by different elements with even higher affinity to oxygen than Ti (for example Y, Ce) as shown before in [35], in order to form more favorable and finer oxide dispersion. Compared to the previous study [15], the oxide contamination was in the present case significantly larger due to use of ethanol wet-milling after dry milling.

It is interesting to point out that, despite the significant contamination by oxides, which is to some extent inherent to the utilized processing route, the mechanical properties were not significantly deteriorated.

With increase in the Ti concentrations, a significant increase in propensity to form ordered intermetallic phases (L_12_ or DO_24_ type) in the FCC matrix phase. At the same time, Ti decreases the melting temperature of the Ni_1,5_Co_1,5_CrFeTi_X_ alloys, due to a eutectic-type of transformation. However, the intermetallic phases predicted by the CALPHAD calculations were not formed even in the SPS-ed materials, or only in the minor fractions (Ti0.7). This occurred due to their slow formation, in combination with lower processing temperature of the PM process and relatively fast cooling after SPS [14].

Table 8 presents the comparison of our tensile results with other materials produced by the same method. Note that there is only a very limited amount of studies showing tensile testing on PM high entropy materials, due to certain problems with preparation of sufficient material volume. Table 8 shows that our results are equivalent, or even superior to other powder metallurgy (PM) HEAs in terms of tensile properties. The full-density Ti0.5A and Ti0.7A alloys even exhibit comparable properties to commercial FCC steels with similar strength levels.

The presented results show that HEAs such as Ni_1,5_Co_1,5_CrFeTi_X_ show a very good tolerance for inclusions and changes of chemical compositions. With increasing use of scrap metal and energy saving technologies aiming at the reduction of the consumption of primary raw materials, such HEAs may play a role in future sustainable metallurgy production [36].

## 5. Conclusions

- The increase in the Ti concentration decreases the average particle size of the powder during mechanical alloying of Ni_1.5_Co_1.5_CrFeTi_X_.
- The thermodynamic calculation with the ThermoCalc TCHEA3 database showed satisfactory prediction accuracy in relation to the obtained experimental data.
- The Ti increases the strength of the Ni_1.5_Co_1.5_CrFeTi_X_ alloys by increasing average atomic size misfit i.e., solid solution strengthening.
- An excellent combination of high strength and ductility can be obtained in Ni_1.5_Co_1.5_CrFeTi_x_ alloys by combining mechanical alloying, SPS and solution annealing.
- The mechanical alloying in ethanol resulted in the formation of oxide dispersion in the SPS-ed bulks.
- Despite the oxide formation, the mechanical properties were not significantly impeded.

## Figures and Tables

**Table 1:** Manufacturers and product identification of used powders.

Element	Manufacturer	Product Identification
Co	Alfa Aesar	Particle size < 44 µm, purity 99.5%, LOT: W08B011
Cr	Alfa Aesar	Particle size < 44 µm, purity 99%, LOT: S18A034
Fe	Aldrich	Particle size 5–9 µm, purity ≥99.5% Lot # MKBS9265V
Ni	Aldrich	Particle size < 50 µm, purity 99.7%, Lot # MKBR6365V
Ti	Alfa Aesar	Particle size < 44 µm, purity 99.5%, LOT: W17A045

**Table 2:** The average sizes of the Ni1.5Co1.5CrFeTix powder particles after mechanical alloying.

Powder	Average Size d [μm]	Deviation [μm]	d_max_ [μm]
Ti0.3	10.68	20.96	249.80
Ti0.5	9.08	15.95	154.36
Ti0.7	8.27	12.70	141.19

**Table 3:** Various measured characteristics of Tix alloys in sintered and annealed state measured by Image and XRD analysis.

Measured Characteristics.	Sintered Alloys			Annealed Alloys		
**Image Analysis**	**Ti0.3**	**Ti0.5**	**Ti0.7**	**Ti0.3A**	**Ti0.5A**	**Ti0.7A**
Porosity [%]	3.77	0.03	0.02	0.43	0.06	0.01
Average grain size [µm]	0.44	1.69	1.14	1.30	1.74	2.13
Average oxide size [nm]	61.80	94.41	87.40	155.54	171.13	171.13
Area of oxides [%]	5.01	5.09	5.03	7.39	8.32	8.29
**XRD Analysis**	**Ti0.3**	**Ti0.5**	**Ti0.7**	**Ti0.3A**	**Ti0.5A**	**Ti0.7A**
Lattice parameter [Å]	3.58	3.58	3.59	3.58	3.59	3.60
FCC phase [%]	95.40	96.40	92.90	96.60	96.50	96.00
Oxides + intermetallics [%]	4.60	3.60	7.10	3.40	3.50	4.00

**Table 4:** Chemical compositions of the respective HEA FCC matrix obtained by EDS analysis of materials in as-sintered and annealed conditions.

	Sintered Alloys		Annealed Alloys	
**Ti0.3**	**Measured Composition [at.%]**	**Target Composition [at.%]**	**Measured Composition [at.%]**	**Target Composition [at.%]**
Co	27.97 ± 0.53	28.3	27.63 ± 0.16	28.3
Cr	17.28 ± 0.44	18.87	19.33 ± 0.12	18.87
Fe	20.11 ± 0.51	18.87	21.09 ± 0.16	18.87
Ni	30.05 ± 0.69	28.3	27.17 ± 0.18	28.3
Ti	4.59 ± 0.83	5.66	4.79 ± 0.61	5.66
**Ti0.5**	**Measured Composition [at.%]**	**Target Composition [at.%]**	**Measured Composition [at.%]**	**Target Composition [at.%]**
Co	27.36 ± 0.70	27.27	26.60 ± 0.22	27.27
Cr	16.82 ± 0.31	18.18	18.46 ± 0.10	18.18
Fe	20.60 ± 1.03	18.18	21.14 ± 0.19	18.18
Ni	28.56 ± 0.34	27.27	26.23 ± 0.26	27.27
Ti	6.66 ± 1.81	9.09	7.58 ± 0.76	9.09
**Ti0.7**	**Measured Composition [at.%]**	**Target Composition [at.%]**	**Measured Composition [at.%]**	**Target Composition [at.%]**
Co	25.90 ± 0.54	26.32	25.57 ±0.47	26.32
Cr	16.95 ± 0.94	17.54	17.90 ± 0.19	17.54
Fe	19.45 ± 1.04	17.54	19.76 ± 0.26	17.54
Ni	27.82 ± 1.78	26.32	25.17 ± 0.77	26.32
Ti	9.88 ± 1.42	12.28	11.60 ± 1.50	12.28

**Table 5:** Results of microhardness testing on the sintered and annealed bulk materials.

Material	Ti0.3	Ti0.5	Ti0.7	Ti0.3A	Ti0.5A	Ti0.7A
Microhardness HV 0.1	448 ± 20.1	524 ± 25.6	556 ± 22.0	355 ± 4.5	379 ± 3.9	500 ± 7.4

**Table 6:** The average results of the tensile tests carried out on Ni_1,5_Co_1,5_CrFeTi_x_ alloys.

Alloy	E (GPa)	R_p0.2_ (MPa)	R_m_ (MPa)	A_t_ (%)	Z (%)	Strain Hardening R_m_–R_p0.2_ (MPa)
Ti0.3A	229.8	781.5	845	2.1	2.8	63.5
Ti0.5A	265.6	930.5	1199	14.1	24.8	268.5
Ti0.7A	228.7	1281.5	1618.5	8.8	12.1	337

**Table 7:** The calculated atomic size difference of produced Ni_1.5_Co_1.5_CrFeTi_X_ alloys in a single-phase state.

Alloy	Ti0.3	Ti0.5	Ti0.7
Atomic size difference δ (%)	4.08	5.03	5.71

**Table 8:** The comparison of mechanical properties of produced Ni_1,5_Co_1,5_CrFeTi_X_ alloys with other similar materials. The values for different PM materials are taken from Refs. [9,37]. The values of wrought steels are taken from https://www.materials.sandvik/cz.

Material	R_p0.2_ (MPa)	R_m_ (MPa)	A (%)
G-Ti0.3	781.5	845	4.4
G-Ti0.5	930.5	1199	11.9
G-Ti0.7	1281.5	1618.5	8.2
Fe30Ni30Co29Mn5.5Cu5.5 + TiC	495	710	11
Fe30Ni30Co29Mn5.5Cu5.5	1125	1276	10
CoCrNi + Boride	1425	1432	1.86
X 7 CrNiAl 17-7—precipitation strengthened	1150	1300	12
SANDVIK 316LVM cold rolled	800	1100	12
X 10 CrNi 18-8 cold rolled	1150	1300	15

## Appendix A

**Table A1:** Chemical compositions of the oxides from Figure A1. The black dots represent oxide particles enriched mostly in Ti. The point EDS analysis cannot be considered as perfectly accurate measurement of chemical composition because even the largest of the oxides are still very small and the EDS signal coming out of them is contaminated by the signal from the Ni, Co, Cr and Fe rich FCC matrix below the particles. Therefore, residual traces of Ni, Co, Cr and Fe are probably a background noise.

Element	Ti0.3	Ti0.3	Ti0.5	Ti0.5	Ti0.7	Ti0.7
	Spectrum 24	Spectrum 25	Spectrum 11	Spectrum 12	Spectrum 1	Spectrum 2
	(at.%)	(at.%)	(at.%)	(at.%)	(at.%)	(at.%)
**O**	**30.21**	**41.46**	**56.89**	**57.20**	**37.6**	**26.51**
**Ti**	**50.50**	**30.95**	**40.55**	**40.11**	**44.9**	**38.98**
Cr	11.83	8.41	0.96	1.01	5.83	8.26
Fe	2.25	5.83	0.71	0.75	4.44	6.95
Co	2.67	6.87	0.33	0.56	4.63	10.01
Ni	2.41	5.65	0.56	0.37	2.61	9.29
